# Efficacy of Dental Bleaching with Whitening Dentifrices: A Systematic Review

**DOI:** 10.1155/2018/7868531

**Published:** 2018-10-30

**Authors:** Bruno G. S. Casado, Sandra L. D. Moraes, Gleicy F. M. Souza, Catia M. F. Guerra, Juliana R. Souto-Maior, Cleidiel A. A. Lemos, Belmiro C. E. Vasconcelos, Eduardo P. Pellizzer

**Affiliations:** ^1^School of Dentistry, University of Pernambuco (UPE), Camaragibe, PE, Brazil; ^2^School of Dentistry, Pernambuco Federal University (UFPE), Recife, PE, Brazil; ^3^School of Dentistry, Dental Materials and Prosthodontics, São Paulo State University (UNESP), São Paulo, Araçatuba, Brazil

## Abstract

A systematic review was performed to evaluate whether whitening toothpastes promote tooth whitening when compared to the use of conventional (nonbleaching) dentifrices. This review was registered at PROSPERO (CRD42017065132) and is based on the Preferred Reporting Items for Systematic Reviews and Meta-Analyses. Electronic systematic searches of PubMed/MEDLINE, Scopus, and the Cochrane Library were conducted for published articles. Only randomized clinical trials in adults that compared the use of so-called whitening dentifrices to the use of nonwhitening dentifrices were selected. The outcome was tooth color change. Twenty-two articles from 703 data sources met the eligibility criteria. After title and abstract screening, 16 studies remained, after which a further five studies were excluded. In total, nine studies were qualitatively analyzed. Significant differences in tooth color change were found between the groups using whitening dentifrices and those using nonwhitening dentifrices. Within the limitations of this study, the evidence from this systematic review suggests that bleaching dentifrices have potential in tooth whitening. However, although many whitening dentifrices have been introduced into the dental market for bleaching treatments, it is important to analyze tooth surface and color changes when performing home bleaching.

## 1. Introduction

Tooth discoloration is one of the most commonly reported complaints in patients seeking aesthetic treatment. Variation in tooth color can be influenced by intrinsic and extrinsic factors, ranging from chemical ingestion to consumption of foods that cause staining [1, 2].

Currently, there are several products on the market that remove stains and claim to whiten teeth. Options range from simple professional prophylaxis and the application of bleaching gels to vital teeth for home use or supervised in a dental office [3]. Bleaching gels normally consist of different concentrations of hydrogen peroxide or carbamide peroxide and involve various forms of application. Furthermore, these different applications result in different mechanisms of activation, which provide dental bleaching through oxi-reduction reactions, based on partial oxidation of the active principle, through which the whitening agent alters the structure of pigment molecules, thus promoting tooth whitening [4, 5].

Several companies have developed bleaching toothpastes, which are considered an alternative to home and/or dental whitening procedures, and which promise bleaching results within 2 to 4 weeks. These toothpastes thus offer increasingly simpler and less costly bleaching methods for those wishing to have whiter teeth [6, 7]. Many of these bleaching toothpastes contain hydrogen peroxide, whereas others contain abrasive components, which promote the removal of extrinsic stains [7, 8].

These abrasives may remove blemishes from the coronary surfaces, giving rise to the idea that alterations in tooth coloration have occurred, which is often used as a marketing strategy by companies to show that teeth are healthy. However, little is known about the efficacy of these bleaching dentifrices compared with conventional (nonbleaching) dentifrices and their effects/alterations on stained teeth regardless of etiology [7, 9, 10].

Therefore, the objective of this systematic review was to evaluate whether whitening toothpastes promote tooth whitening when compared to the use of nonbleaching dentifrices. The hypothesis of the study is that bleaching dentifrices do not promote tooth whitening.

## 2. Materials and Methods

### 2.1. Protocol Registration

The current systematic review was performed following the Preferred Reporting Items for Systematic Reviews and Meta-Analyses. The methods used in this review are registered on PROSPERO (CRD42017065132).

### 2.2. Research Methods

The selection of articles was performed individually by two authors (Bruno G. S. Casado and Cleidiel A. A. Lemos) using published papers found in the Cochrane Library, PubMed/MEDLINE, and Scopus databases from inception to December 2017. The following terms were used in the search strategy: “tooth bleaching and dentifrice OR dental bleaching and dentifrice OR tooth bleaching and toothpaste OR dental bleaching and toothpaste.”

Two researchers also manually searched for papers published up to December 2017 in specific journals such as Dental Materials and Journal of Dentistry and Operative Dentistry. A third author (Gleicy F. M. Souza) determined divergences in paper selection by the researchers and a consensus was obtained through discussion.

### 2.3. Eligibility Criteria

The selection criteria included randomized clinical trials (RCTs) and articles published in English. The exclusion criteria included prospective and retrospective studies, crossover studies, *in vitro* studies, animal studies, mechanical studies, case reports, and literature reviews.

### 2.4. Search Strategy

Clinical studies were selected from the title and abstract through electronic searches conducted by two independent researchers. In studies where it was not possible to obtain sufficient information, the complete article was downloaded. After reading the title and abstract, the studies that did not meet the inclusion criteria were excluded.

The following specific question was elaborated based on the population, intervention, control, and outcomes criteria: “Do bleaching dentifrices effectively promote tooth whitening?” According to these criteria, the population was composed of patients who used dentifrices, and the intervention was the use of so-called whitening dentifrices compared with the use of nonwhitening dentifrices. The evaluated outcome was the efficacy of bleaching dentifrices on tooth color change.

### 2.5. Risk of Bias and Evaluation of Study Quality

Two investigators (Bruno G. S. Casado and Cleidiel A. A. Lemos) evaluated the methodological quality of the included studies using bias analyses based on the Cochrane criteria for assessing the risk of bias. This tool assessed the quality and risk of bias of the included studies based on sequence generation, allocation concealment, blinding of participants, personnel or outcome investigator, incomplete outcome data, selective outcome reporting, and other sources of bias and was rated as low/high or unclear risk of bias according to the studies evaluated.

### 2.6. Data Collection and Analysis

The data collected from the articles were classified as quantitative and qualitative by one researcher (Bruno G. S. Casado) and then verified by another researcher (Gleicy F. M. Souza). All disagreements were resolved by a third researcher (Cleidiel A. A. Lemos) through discussion until a consensus was reached. Quantitative and qualitative data were tabulated to aid the comparison.

### 2.7. Additional Analysis

An additional analysis was performed using the kappa coefficient, which was calculated to establish the interexaminer agreement in study selection from the three databases. The kappa value was obtained by evaluating the titles and abstracts selected. The Cochrane Library (*K* = 0.94), PubMed/MEDLINE (*K* = 0.71), and Scopus (*K* = 0.92) showed a high level of agreement.

## 3. Results

**Figure 1:**
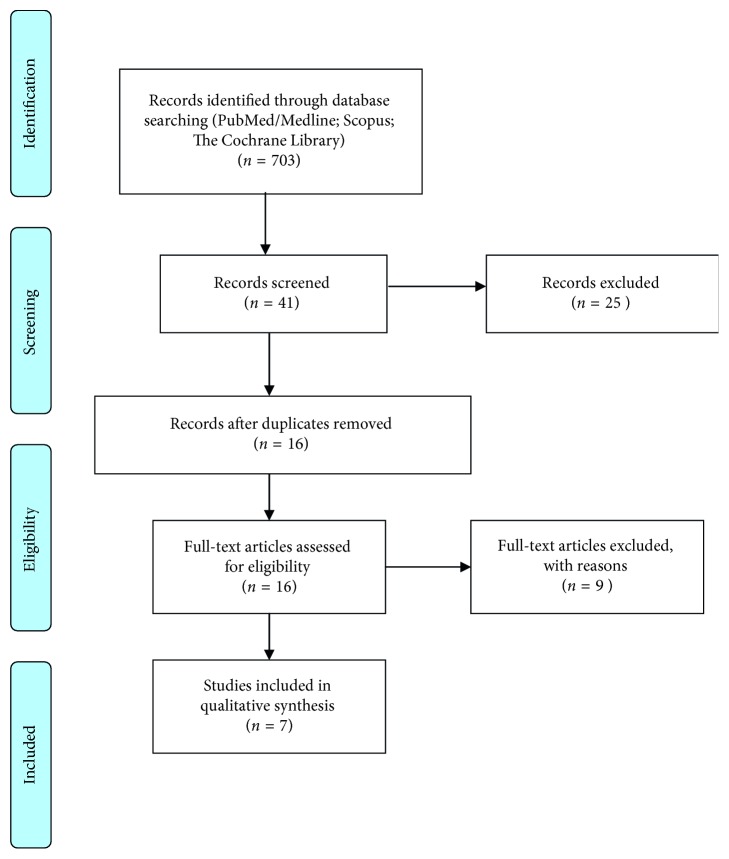
Flow chart showing the steps in the literature search.

The database search identified a total of 703 articles, 287 of which were from PubMed/MEDLINE, 303 from Scopus, and 113 from the Cochrane Library. After removal of duplicate references and a thorough review of titles and abstracts, 16 studies were read in full. After reading, nine studies were excluded (Table 1). Details regarding the search strategy are presented in the flow diagram (Figure 1).

In total, seven studies were selected for qualitative analyses and are summarized in Table 2. All selected studies were RCTs published between 2001 and 2016. A total of 1,399 patients with a mean age of 36.89 years were included in the studies, of which 879 used some type of dentifrice considered to be a bleaching agent by the manufacturer. The groups of patients evaluated varied according to the dentifrice, and the effectiveness of nine products was tested: Arm & Hammer® Advance White® Extreme Whitening Baking Soda and Peroxide Toothpaste (*n*=86), Arm & Hammer® Truly Radiant Toothpaste (*n*=59), Crest® 3-D white radiant mint toothpaste (*n*=56), Crest® Extra whitening (*n*=363), Colgate® Simple White® Advanced Whitening Toothpaste Sparkling Mint (*n*=21), Colgate® Baking Soda Peroxide (*n*=216), Colgate Luminous White® (*n*=32), Close-Up White Now® (*n*=31), and Oral B 3D white (*n*=15).

The bleaching effectiveness of the studied dentifrices was evaluated. Four studies showed that the products evaluated were effective in bleaching teeth using the VITA color scale (subjective method), and three studies showed that the products were effective in bleaching teeth using spectrophotometry (objective method).

In relation to daily brushing frequency, most studies [20–24] reported that using toothpaste twice a day increased tooth whitening, but two studies indicated that patients who brushed three times a day were more likely to have whiter teeth. In addition, the follow-up period ranged from 5 days to 8 weeks, with the most common period being 4 weeks (six studies). All studies included in this review examined subjects who used conventional toothpaste (not considered to have a bleaching effect by the manufacturers) as the control group.

**Figure 2:**
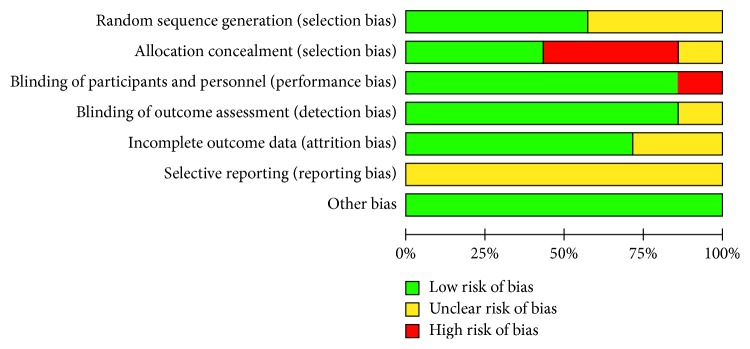
Cochrane scale for bias risk.

The relevance of the articles included in this systematic review was considered satisfactory as all studies were RCTs with a low risk of bias (Figure 2). We included Cochrane randomized clinical trial studies to determine the bias scale in each study. All included studies were characterized as double-blind (randomized studies were performed with the patient and the evaluator blinded to the product), which is considered important in understanding responses in experimental clinical research.

### 3.1. Bleaching Effectiveness

In the four studies that evaluated tooth color change using the VITA shade guide, all studies [20, 22–24] reported that bleaching dentifrices were effective for tooth whitening when compared with regular dentifrices (*p* < 0.05). However, Gerlach et al. [20] found differences between the groups only in the evaluation period following 4 weeks of dentifrice use.

In the studies that used a spectrophotometer to analyze tooth whitening, two studies [21, 25] demonstrated that bleaching dentifrices differed in relation to conventional dentifrices. However, Horn et al. [25] tested three different dentifrices, and found that only the use of Colgate Luminous White® had a tooth-whitening effect after 2 weeks. In agreement, a study by Pintado-Palomino et al. [7] showed that most bleaching and control dentifrices showed similar clinical performances, without a significant chromatic reduction, during a 4-week follow-up period.

## 4. Discussion

The results of this systematic review indicate that most of the included studies showed a significant change in dental coloration following the use of commercially available bleaching dentifrice agents when used for a period of between 5 days and 8 weeks.

Although peroxide bleaching materials are well-established for aesthetic tooth whitening, the use of these substances in dentifrices is quite limited [25]. In addition to causing alterations in products, high concentrations of hydrogen peroxide need to be counterbalanced by the use of soft tissue protective barriers in order to maintain contact with dental surfaces, which is not the case with bleaching toothpastes [8].

However, Isaacs et al. [21], Kakar et al. [22], and Ghassemi et al. [23] observed that the presence of 1.0% hydrogen peroxide in the chemical formulation of dentifrices caused tooth coloration changes when compared with nonwhitening toothpastes. These findings agreed with those obtained by Sharma et al. [17] who demonstrated the bleaching potential of these dentifrices and concluded that the presence of hydrogen peroxide was able to significantly interfere with dental chromatic alterations over a brushing period of 2 to 6 weeks. Therefore, it is important to consider the concentration of hydrogen peroxide and its contact time as important contributors to effective tooth whitening [19].

On the other hand, studies by Gerlach et al. [20], Horn et al. [25], and Ghassemi et al. [24] tested bleaching dentifrices that were free from any type of peroxide and obtained satisfactory results regarding changes in tooth coloration during the use of these toothpastes. These results may have been due to the presence of high performance abrasive agents contained in the bleaching dentifrices such as silica, which was present in almost all products included in this review [7, 21–25]. These abrasive agents promote the gradual physical removal of extrinsic pigments without effectively whitening teeth. Therefore, these bleaching dentifrices are sometimes considered only as surface spot removers [7, 8, 25].

Interestingly, the studies by Hilgenberg et al. [26] and Özkan et al. [27] showed that bleaching dentifrices promoted morphological changes on the surfaces of tooth enamel. Therefore, it is important to consider that brushing with toothpastes containing abrasive substances should be done with caution, as the indiscriminate use of dentifrices with large quantities of these agents can lead to irreversible damage of hard dental tissues and restorative materials; they can also lead to recession of the gingiva, abrasion in the cervical region, and in some cases, dentin hypersensitivity [8, 26].

Another limiting factor is that these studies did not use similar evaluation methods, making it difficult to compare the parameters studied in the coloration change analyses in this review. Dozic et al. [28] proposed that the spectrophotometer was the most reliable instrument on the market for tooth coloration analysis; the accuracy of the results is related to the positioning of the equipment at the same point of the dental surface at all times of analysis [14].

However, among the studies selected in this review which used spectrophotometry as the evaluation method, only the study by Isaacs et al. [21] demonstrated a significant color change following use of the bleaching dentifrices. This probably occurred because the products tested by Isaacs et al. [21] contained hydrogen peroxide associated with a high performance silica. These results are in agreement with those by Sharma et al. [17], who confirmed that these two substances in a dentifrice were able to remove extrinsic stains, reducing the yellow color pigmentation (*b*^*∗*^ parameter) of the teeth, when compared to conventional dentifrices.

The findings of Horn et al. [25] and Pintado-Palomino et al. [7], who also used spectrophotometry as a method of evaluation, did not show a significant difference between bleaching and conventional dentifrices, a factor justified by the absence of hydrogen peroxide in the dentifrices tested. Although in the study by Horn et al. [25], a statistical difference was shown in one of the test groups (Colgate Luminous White dentifrice) by altering the values of *L*^*∗*^ (brightness), it was also seen that according to the NBS criterion, the value of Δ*E* was 1.15, which meant a change in color was not perceived by the human eye. This change in luminosity probably occurred due to the presence of abrasive contents in this dentifrice, such as hydrated silica.

The four studies that used the VITA shade guide observed a statistically significant difference between the test and control groups. These favorable findings may have been attributed to the method used to analyze the color on the total surface of the tooth, without taking into account specific points [14].

The limitations of the present systematic review include the following: the lack of clinical studies using the same method to evaluate the color of dental substrates, in order to allow a comparison of the parameters included in the data analysis; the lack of studies that took into account the durability of color change following discontinuation of the bleaching dentifrices; and the studies did not take into account the frequency of brushing with bleaching dentifrices, as brushing time can influence color change^.^ [7]. Thus, future studies are necessary in order to investigate characteristics such as morphological alterations of the dental surface caused by dentifrice materials, in order to establish an effective time-of-use protocol, the influence of dentifrice components on whitening properties, and the durability of tooth whitening after whitening toothpaste is discontinued.

## 5. Conclusions

Within the limitations of this study, the evidence from this systematic review suggested that bleaching dentifrices have potential in tooth whitening. However, these results should be interpreted with caution before any decision is made, and more randomized clinical trials are required to better determine the efficacy of bleaching dentifrices due to their possible morphological alterations of dental tissues.

## Figures and Tables

**Table 1:** Reasons for exclusion of “9” articles.

Author, year	Reason for exclusion
Llena et al. 2016 [11]	Use of gel substance associated with dentifrice for enzymatic activation.
Motta et al. 2013 [12]	Abstract only
Forner et al. 2012 [13]	Use of gel substance associated with dentifrice for enzymatic activation.
Raoufi and Birkhed, 2010 [14]	Another method of analysis
Collins et al. 2008 [15]	Crossover study
Yhudira et al. 2007 [16]	Association of different bleaching methods.
Sharma et al. 2004 [17]	Without control group
Soparkar et al. 2004 [18]	Without control group
Gerlach et al. 2004 [19]	Without conventional toothpaste as a control group

**Table 2:** Summary of characteristics of included studies.

Author/year	Design of study	Patients, *n*	Mean age (range)	Frequency of use (time)	Evaluation time	Evaluation methods	Groups, *n*	Color mean (SD): Reduction in score			Difference between groups	Effect of whitening dentifrice
								Follow-up period	Whitening dentifrice	Control group		
Ghassemi et al. 2012 [23]	Randomized controlled trial	135	38,9 (19–70)	Twice daily (1 minute)	4 and 6 weeks	Shade guide	*G1: Arm & Hammer advanced white* (*n*=86) G2: Crest cavity protection toothpaste (*n*=49)	Week 4	(G1) 1.82 (0.80)	(G2) 0.07 (0.42)	G1^*∗*^ × G2 *p* < 0.0001	Positive
								week 6	(G1) 2.57 (0.99)	(G2) −0.04 (0.69)	G1^*∗*^ × G2 *p* < 0.0001	Positive

Ghassemi et al. 2015 [24]	Randomized controlled trial	178	38,5 (18–75)	Twice daily (2 minutes)	5 days and 2, 4, and 6 weeks	Shade guide	*G1: Truly radiant toothpaste* (*n*=59) *G2: Crest 3D white radiant mint toothpaste* (*n*=56) G3: Colgate cavity protection toothpaste (*n*=63)	Day 5	(G1) 0.597	(G3) −0.08	G1^*∗*^ × G2 *p*=0.0105	Positive
									(G2) 0.324		G1/G2^*∗*^ × G3 *p* < 0.0001	
								week 2	(G1) 1.172	(G3) 0.046	G1 × G2 *p*=0.1595	Positive
									(G2) 0.837		G1/G2^*∗*^ × G3 *p* < 0.0001	
								week 4	(G1) 1.170	(G3) 0.107	G1 × G2 *p*=0.2409	Positive
									(G2) 1.326		G1/G2^*∗*^ × G3 *p* < 0.0001	
								week 6	(G1) 2.081	(G3) 0.038	G1^*∗*^ × G2 *p*=0.0383	Positive
									(G2) 1.467		G1/G2^*∗*^ × G3 *p* < 0.0001	

Gerlach et al. 2001 [20]	Randomized controlled trial	278	43,9 (19–79)	Twice daily (NI)	4 and 8 weeks	Shade guide	*G1: crest extra whitening* (*n*=144) G2: Arm & Hammer dental care dentifrice (*n*=134)	Week 4	(G1) 1.04	(G2) 0.53	No difference	None
								week 8	(G1) 1.42	(G2) 0.96	G1^*∗*^ × G2 *p* < 0.05	Positive

Isaacs et al. 2001 [21]	Randomized controlled trial	654	43 (NI)	Twice daily (NI)	4 and 8 weeks	Spectroscopy	*G1: Crest extra whitening (silica)* (*n*=219) *G2: Colgate baking soda and peroxide* (*n*=216) G3: Crest cavity protection (*n*=219)	Week 4	(G1) 0.05	(G3) −0.14	G1^*∗*^ × G3 *p*=0.007	Positive
									(G2) 0.08		G2^*∗*^ × G3 *p*=0.002	Positive
								week 8	(G1) 0.03	(G3) −0.25	G1^*∗*^ × G3 *p* < 0.001	Positive
									(G2) 0.10		G2^*∗*^ × G3 *p* < 0.001	Positive
											G1 × G2 No difference	

Kakar et al. 2004 [22]	Randomized controlled trial	44	34,15 (NI)	Twice daily (2 minutes)	2 weeks and 4 weeks	Shade guide	*G1: Colgate simply white* (*n*=21) G2: control dentifrice (*n*=23)	Week 2	(G1) 4.04 (1.40)	(G2) 0.41 (0.55)	*p* < 0.05 G1	Positive
								week 4	(G1) 5.17 (1.09)	(G2) 0.53 (0.63)	*p* < 0.05 G1	Positive

Horn et al. 2014 [25]	Randomized controlled trial	60	NI (19–36)	Three times a day (2-3 minutes)	2 weeks	Spectroscopy	G1: Colgate total 12 (*n*=15) *G2: Close-Up white now* (*n*=15) *G3: Oral B 3D white* (*n*=15) *G4: Colgate luminous white* (*n*=15)	Week 2	(G2) −0.7	(G1) −1.1	G1 × G2 or G3 No difference	None
									(G3) −0.3			
									(G4) −1.7			
											G4^*∗*^ × G1 *p*=0.01	Positive

Pintado-Palomino et al. 2016 [7]	Randomized controlled trial	50	22,9 (19–36)	Three times a day (2-3 minutes)	4 weeks	Spectroscopy	*G1: Colgate luminous white* (*n*=17) *G2: Close-Up white now* (*n*=16) G3: Sorriso (*n*=17)	Week 4	(G1) 5.1 (2.8)	(G3) 4.4 (3.0)	No difference	None
									(G2) 6.8 (3.5)			

^*∗*^Groups with significant statistical difference. NI, not informed.
